# Survival analysis and mortality predictors of COVID-19 in a pediatric cohort in Mexico

**DOI:** 10.3389/fpubh.2022.969251

**Published:** 2022-12-16

**Authors:** Fortino Solórzano-Santos, América Liliana Miranda-Lora, Horacio Márquez-González, Miguel Klünder-Klünder

**Affiliations:** ^1^Unidad de Investigación en Enfermedades Infecciosas, Hospital Infantil de México Federico Gómez, Instituto Nacional de Salud, Ciudad de México, Mexico; ^2^Unidad de Investigación Epidemiológica en Endocrinología y Nutrición, Hospital Infantil de México Federico Gómez, Instituto Nacional de Salud, Ciudad de México, Mexico; ^3^Departamento de Investigación Clínica, Hospital Infantil de México Federico Gómez, Instituto Nacional de Salud, Ciudad de México, Mexico; ^4^Subdirección de Investigación, Hospital Infantil de México Federico Gómez, Instituto Nacional de Salud, Ciudad de México, Mexico

**Keywords:** SARS-CoV-2, comorbidity, COVID-19, mortality, pediatrics, Mexico

## Abstract

**Background:**

The new coronavirus SARS-CoV-2 pandemic has been relatively less lethal in children; however, poor prognosis and mortality has been associated with factors such as access to health services. Mexico remained on the list of the ten countries with the highest case fatality rate (CFR) in adults. It is of interest to know the behavior of COVID-19 in the pediatric population. The aim of this study was to identify clinical and sociodemographic variables associated with mortality due to COVID-19 in pediatric patients.

**Objective:**

Using National open data and information from the Ministry of Health, Mexico, this cohort study aimed to identify clinical and sociodemographic variables associated with COVID-19 mortality in pediatric patients.

**Method:**

A cohort study was designed based on National open data from the Ministry of Health, Mexico, for the period April 2020 to January 2022, and included patients under 18 years of age with confirmed SARS-CoV-2 infection. Variables analyzed were age, health services used, and comorbidities (obesity, diabetes, asthma, cardiovascular disease, immunosuppression, high blood pressure, and chronic kidney disease). Follow-up duration was 60 days, and primary outcomes were death, hospitalization, and requirement of intensive care. Statistical analysis included survival analysis, prediction models created using the Cox proportional hazards model, and Kaplan-Meier estimation curves.

**Results:**

The cohort included 261,099 cases with a mean age of 11.2 ± 4 years, and of these, 11,569 (4.43%) were hospitalized and 1,028 (0.39%) died. Variables associated with risk of mortality were age under 12 months, the presence of comorbidities, health sector where they were treated, and first wave of infection.

**Conclusion:**

Based on data in the National database, we show that the pediatric fatality rate due to SARS-CoV-2 is similar to that seen in other countries. Access to health services and distribution of mortality were heterogeneous. Vulnerable groups were patients younger than 12 months and those with comorbidities.

## Introduction

Severe acute respiratory syndrome coronavirus 2 (SARS-CoV-2) emerged in China at the end of 2019, has spread worldwide, and Coronavirus disease 2019 (COVID-19) was declared a pandemic by the World Health Organization (WHO) in March 2020 (1). As of March 21, 2022, more than 471,925,910 COVID-19 cases have been confirmed worldwide, with 6,104,420 deaths reported. In Mexico, 5,633,928 cases and 322,072 deaths have been reported (2, 3). Reported disease burden and case fatality rates vary considerably among different age groups during the different waves of the pandemic, and COVID-19 in children is thought to typically cause mild-to-moderate disease. Additionally, while fewer children appear to develop serious sequelae such as multisystem inflammatory syndrome (MIS-C) (4), some reports state that the age group with higher mortality is infants <1 year old (5, 6). The pediatric population accounts for 1–2% of the total cases; however, this number varies in the different waves of the pandemic, as reported by Rovida et al. (7). Approximately 6% of all infected children develop severe illness that requires pediatric intensive care unit (ICU) admission, and similar to adults, hospitalized patients, especially critically ill patients, have pre-existing underlying diseases or comorbidities (7). There appears to be an effect of age and comorbidities on the prognosis of children with COVID-19, according to a recent meta-analysis those <1 year old were more likely to be admitted to ICU, Odds Ratio (OR) = 1.63 (95% CI: 1, 40 to 1.90); and death, OR = 2.08 (95% CI: 1.57 to 2.86) and the odds of death increased among children aged 10–14 years OR = 2.15 and >14 years OR = 2.15. A meta-analysis showed a more significant association with outcomes (ICU and death) when comparing patients with and without comorbidities. For example, with an added disease, the OR was 1.49 for ICU admission and 2.34 for death; with three comorbidities, it increased to 4.32 and 5.56, respectively (8).

Using national open data and information from the Ministry of Health, Mexico, this cohort study aimed to identify clinical and sociodemographic variables associated with COVID-19 mortality in pediatric patients (3). Risk factors for severe disease and death in the pediatric population were analyzed, to identify those groups that should be priority for vaccination and in those that require other interventions to the risk reduction. It was made an analysis about if the attention in some sector of our health system influence over the patient's prognosis.

## Methods

In Mexico, by official provision, hospitals of the health system that receive patients with suspected or confirmed SARS-CoV-2 virus infection have to register patient data in a public database created by the General Directorate of Epidemiology and contains the information of all public and private institutions in the country that treated hospitalized patients and that reported to the Ministry of Health of Mexico through the SISVER platform (for its acronym in Spanish, Epidemiological Surveillance System for Respiratory Diseases). Using this database (see Data Availability Statement), we analyzed data of a cohort of pediatric patients (under 18 years of age) from April 14, 2020, to January 16, 2022. The selection criteria applied were any gender and a positive PCR test for SARS-CoV-2 or antigen test plus positive criteria for COVID-19, as issued by a ruling committee. Cases with inconsistencies in the dates of diagnosis, hospitalization and death were excluded. Time zero was defined as the date of diagnostic confirmation, and follow-up was recorded up to day 60. Primary outcome was death related to COVID-19.

Risk factors evaluated were age group (according to the American Academy of Pediatrics (9): <12 months, 1–3, 3.1–5, 5.1–12, and 12.1–18 years), states of residence (Mexico is divided into 32 states), health system to which they had access (Mexican Institute of Social Security, IMSS; Institute of Security and Social Services for State Workers, ISSSTE; Ministry of Health, SSA; Private Hospitals and others such as well-being Regime, University Hospitals, Secretary of National Defense, Secretary of the Navy).

The contagion rates were divided into the following periods: first wave, from the first case in Mexico (April 2020) to September 30, 2020; the second wave, from October 1 to May 14, 2021; the third wave, from May 15 to December 3, 2021, and the fourth wave, from January 16, 2022, to the current date.

Other risk variables included were the presence of comorbidities such as obesity, diabetes, asthma, cardiovascular disease, immunosuppression, arterial hypertension, chronic kidney disease, and the need for mechanical ventilation.

Hospitalization, the complication of COVID-19 with pneumonia, and the need for intensive care were identified as confounding variables.

### Statistical analysis

Qualitative variables are presented as frequencies and percentages, age (in years) is presented as a parametric distribution and is expressed as mean and standard deviation. Outcomes variables, namely, hospitalization, ICU requirement, and lethality, were estimated using the formula (10): outcome patient's number/number of patients exposed per 100 or 1,000 infected subjects by State and by sector of Health. The Chi-squared test was used to demonstrate differences in the variables between survivors vs. non-survivors. For the prediction models, the Cox proportional hazards with *forward stepwise* variable selection method was used, and variables entered into the multivariate models based on biological plausibility and initial significance level of *p* < 0.1 in the bivariate analysis. Models were created for mortality in the entire infected population, hospitalization, pneumonia, and ICU admission. Internal validation of each model was performed by calculating each patient's probability in the cohort with the formula (11), λ(*t*) = λ_0_(*t*) exp(β^*T*^*X*), in which, λ = hazard function; t = failure time of interest; and, β^*T*^*X* = It represents the probability of survival in time t of the subjects with a certain pattern of X values in the explanatory variables and by estimating the value of the area under the curve with death as the variable. Kaplan-Meier survival curves were created, and statistical significance was calculated using the Log-Rank test. All statistical analyses used SPSS (*version* 25, for MAC) and STATA *version* 16.

## Results

**Figure 1:**
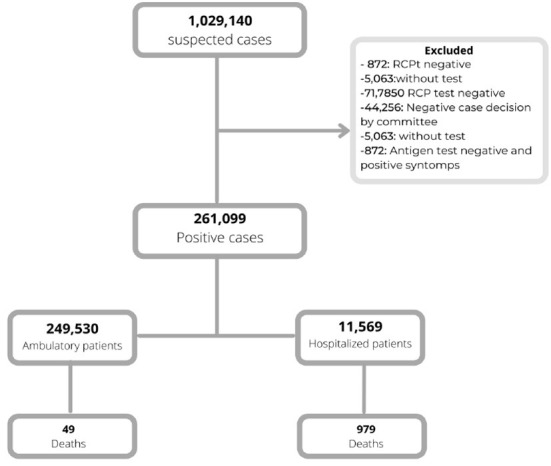
Selection of the cohort of pediatric patients with SARS-CoV-2.

COVID-19 was suspected in 1,029,140 children and infection by SARS-CoV-2 was confirmed in 261,099. Mean age of the cohort was 11.2 ± 4 years, 131,045 (50.4%) were males, and 11,569 (4.43%) were hospitalized while 249,530 (95.5%) were ambulatory patients (Figure 1). Infections were the highest (50.8%) in children aged > 12.1 years while those aged < 12 months accounted for 5.6% of all infections.

**Figure 2:**
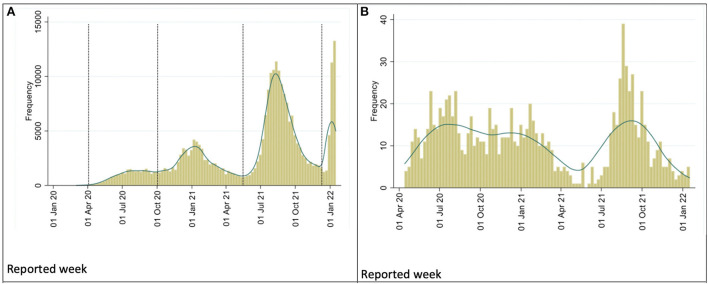
**(A)** Frequency of SARS-CoV-2 infections in the pediatric population in Mexico. **(B)** Frequency of deaths of pediatric patients due to SARS-CoV-2 Mexico.

Figure 2A shows the cumulative 7-day incidence rate of confirmed COVID-19 cases from April 2020 to January 2022.Fourth waves are visible in this chart. Those with the highest number correspond to the third (dominant delta variant) and fourth wave (dominant omicron variant). However, during the first and second waves caused the most deaths, as shown in Figure 2B.

There were 1,028 deaths (0.39%); of these, 49 (0.02%) were among ambulatory patients and 979 (8.46%) in hospitalized patients. Thus, hospitalized patients accounted for 95.2% of all deaths. The global case fatality rate (CFR) was 3.9% with an incidence density of deaths of 6.8 patients per 100,000 days. Lethality was more frequent in children < 12 months old (CFR 2.76) and those aged 1–3 years (CFR 0.60). Next, 33.1% of non-survivors presented with one or more comorbidities; this value was 6.7% among survivors, and the CFR was 2 (per 100 patients). The comorbidities with highest CFR were chronic kidney disease, cardiovascular disease, immunosuppression, and hypertension. Patients requiring mechanical ventilatory assistance had the highest CFR (Table 1).

**Table 1:** General characteristics of pediatric patients with SARS-CoV-2 and the differences between survivors and non-survivors.

	**Total** ***n*** = **261,099**		**Survivor** **260,071**		**No survivor** **1,028**		Case fatality rate^*^
	***n***	**percentage**	***n***	**percentage**	***n***	**percentage**	
**Gender**							
Female	130054	49.80%	129575	49.80%	479	46.60%	0.37
Male	131045	50.20%	130496	50.20%	549	53.40%	0.42
**Group age**							
<12 months	14660	5.60%	14255	5.50%	405	39.40%	2.76
1–3 years	12484	4.80%	12409	4.80%	75	7.30%	0.6
3.1–5 years	13936	5.30%	13894	5.30%	42	4.10%	0.3
5.1–12 years	87357	33.50%	87166	33.50%	191	18.60%	0.22
12.1–18 years	132662	50.80%	132347	50.90%	315	30.60%	0.24
**Type patient**							
Ambulatory	249530	95.60%	249481	95.90%	49	4.80%	0.02
Hospitalized	11569	4.40%	10590	4.10%	979	95.20%	8.46
**Wave of infections**							
1st wave	1195	0.50%	1142	0.40%	53	5.20%	4.43
2nd wave	47850	18.30%	47389	18.20%	461	44.80%	0.96
3rd wave	180915	69.30%	180415	69.40%	500	48.60%	0.27
4st wave	31139	11.90%	31125	12.00%	14	1.40%	0.04
**Health care services**							
I.M.S.S	69689	26.70%	69289	26.60%	400	38.90%	0.57
I.S.S.S.T.E	3784	1.40%	3745	1.40%	39	3.80%	1.03
S.S.A	176812	67.70%	176272	67.80%	540	52.50%	0.31
Private hospital	7365	2.80%	7350	2.80%	15	1.50%	0.2
Other	3449	1.30%	3415	1.30%	34	3.30%	0.99
**Commorbilities**							
Diabetes	1121	0.40%	1069	0.40%	52	5.10%	4.64
Immunosupression	1243	0.50%	1153	0.40%	90	8.80%	7.24
Pulmonary disease	6711	2.60%	6692	2.60%	19	1.80%	0.28
Hypertension	904	0.30%	852	0.30%	52	5.10%	5.75
Obesity	7178	2.80%	7088	2.70%	90	8.80%	1.25
Cardiovascular disease	952	0.40%	883	0.30%	69	6.70%	7.25
Chronic kidney disease	500	0.20%	456	0.20%	44	4.30%	8.8
**Outcomes**							
Pneumonia	5997	2.30%	5313	2.00%	684	66.50%	11.41
Mechanical ventilatory assistence	914	0.40%	505	4.80%	409	42.00%	44.75
Intensive care unit	1156	0.40%	879	8.40%	277	28.40%	23.96

* Case Fatality rate by 100 patients with SARS-CoV-2.

Analysis of outcomes, classified according to the health care institution used, showed that the lowest CFR was recorded in private hospitals (0.1 per 1,000 patients with SARS-CoV-2) even though rate of hospitalization was 3.5 per 100 infected patients. The SSA treated 54.8% of all hospitalized patients and 20% of patients treated in the country's ICUs. Data on other health care institutions are shown in Table 2.

**Table 2:** Comparison of outcomes according to the health institutions that treated pediatric patients with SARS-CoV-2.

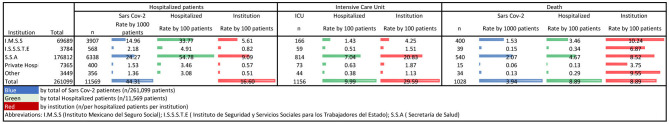

**Figure 3:**
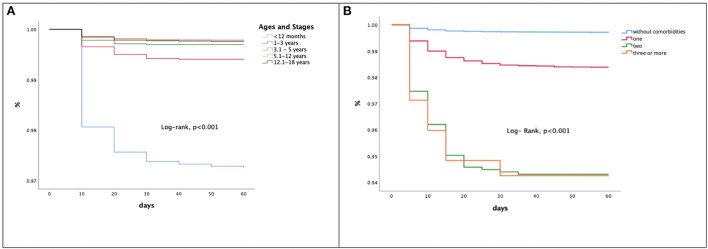
**(A)** Survival analysis of patients with SARS-CoV-2 by age groups. **(B)** Comparative survival function in pediatric patients with SARS-CoV-2 by comorbidities.

Data analysis stratified according to the 32 states of the Country showed that a majority of infections and hospitalization occurred in Mexico City (192.8 per 1,000 patients hospitalized). However, the highest CFR was recorded in other states and was related to access to ICU care (Supplementary Table S1). Kaplan-Meier univariate survival analysis showed that the age group with the lowest survival was children under 12 months of age (Figure 3A), and that the sum of comorbidities was associated with a lower probability of survival (Figure 3B).

Table 3 shows mortality in the total population with SARS-CoV-2 and stratified mortality analysis by prediction models, namely, hospitalized, pneumonia and ICU patients. All models included age (years), comorbidities, health sector, waves of contagion, and fatality rate by state as variables. We found that predictive capacity decreased in patients with pneumonia (AUC = 0.56) and ICU admission (AUC = 0.32). Age in years, cardiovascular disease, and chronic kidney disease were the variables most related to death in all models.

**Table 3:** Predictives models of mortality in pediatrics patients with SARS-CoV-2.

	**Beta**	**HR**	**95%-CI**	
**Total of patients with positive test**				
Age (years)	−0.067	0.935	0.925	0.945
Diabetes	0.697	2.007	1.438	2.802
Immunosuppression	1.066	2.905	2.311	3.652
Asthma	−1.81	0.627	0.44	0.7
Hypertension	0.758	2.134	1.514	3.009
Obesity	0.656	1.927	1.519	2.445
Cardiovascular disease	1.099	3.001	2.323	3.878
Renal chronic disease	1.247	3.479	2.51	4.824
Pneumonia	3.882	48.506	41.948	56.089
Hospitalized	5	124.2	106	145.2
Fatality rate state >10 by 1000 patients	0.453	1.572	1.089	2.271
First wave	0.149	1.16	1.03	1.3
AUC = 0.89				
**Hospitalized**				
Age	−0.12	0.887	0.877	0.897
First wave	4.016	55.455	30.505	100.812
Second wave	3.02	20.488	11.994	34.997
Diabetes	−1.109	0.33	0.227	0.479
Immunosuppression	1.748	5.743	4.514	7.307
Hypertension	−0.617	0.539	0.366	0.796
Obesity	0.887	2.429	1.901	3.104
Cardiovascular disease	1.602	4.96	3.733	6.591
Renal chronic disease	1.393	4.026	2.738	5.92
Fatality rate state>10 per 1000 patients	−1.87	2.5	1.2	3.4
Asthma	1.763	0.652	0.542	0.783
I.S.S.S.T.E	−0.667	0.513	0.358	0.735
S.S.A	−1.206	0.299	0.162	0.554
AUC = 0.62				
**Pneumonia**				
Age	−0.031	0.97	0.958	0.982
First wave	2.995	19.994	8.408	47.543
Second wave	2.495	12.119	5.394	27.225
Third wave	1.96	7.099	3.163	15.933
Diabetes	−0.445	0.641	0.443	0.928
Immunosuppression	−0.563	0.57	0.436	0.744
Hypertension	−0.83	0.436	0.299	0.635
Obesity	−0.369	0.691	0.526	0.909
Cardiovascular disease	−0.891	0.41	0.304	0.553
Renal chronic disease	−1.145	0.318	0.223	0.454
Asthma	−1.34	0.765	0.564	0.821
AUC = 0.56				
**ICU**				
Age	0.019	1.019	1	1.039
Cardiovascular disease	0.844	2.326	1.553	3.483
Renal chronic disease	0.671	1.956	1.02	3.75
AUC = 0.32				

All models included the next variables: age (years), comorbidities, health sector, waves of contagion, and fatality rate by state.

## Discussion

The emergence of COVID-19 has required rapid and continuous learning to face the challenges that this pandemic has presented. Concepts around SARS-CoV-2 that were prevalent at the start of the pandemic have changed; hence, frequent revision of the global outlook remains highly relevant. Although COVID-19 has mainly affected the adult population, it has also spread to the pediatric population, albeit to a lesser extent. Thus, identification of risk factors associated with mortality in the pediatric population is relevant as it can support decision-making aimed at prevention, care, and restoration of the damage caused by the disease.

In Mexico, the frequency of pediatric COVID-19 cases in the different waves was consistent with school closures and social distancing. Furthermore, Rovida et al. (7) reported a lower intensity of SARS-CoV-2 exposure was seen at the start of the pandemic. Additionally, the transition from lockdown to the reopening of schools coincided with an increase in the number of cases in the third and fourth waves. The change in frequency of infection in the recent months is related to the appearance of new variants with greater transmissibility, immune escape, and diagnostic failures (12). Nevertheless, CFR has decreased, which was much higher in the first wave compared to subsequent outbreaks (Table 1). This could be related to the saturation of health services prior to the opening of emergency medical units at the beginning of the pandemic as well as the steep learning curve for the care of COVID-19 patients.

Worldwide, more than 90% of pediatric patients were either asymptomatic or had mild-to-moderate disease (13) with low risk of mortality (14). Our results are consistent with this profile as more than 95% of the positive cases in our cohort exhibited satisfactory outcomes and did not require hospitalization. Moreover, compared to data in our previous report on trends in adults, frequency of hospitalization in Mexican children was 5 times lower, need for intubation was 43-fold less, admission to intensive care was 21 times lower, and mortality was 25-fold less (15).

Factors that can potentially explain the differences in COVID-19 severity between children and adults include immune response, angiotensin converting enzyme 2 distribution (limiting infection), androgen's transmembrane protease serine 2-mediated actions that might result in fewer fatalities in prepubertal children, observed divergence between the sexes in SARS-CoV-2 infection, age-related increase in endothelial damage and changes in clotting function, higher prevalence of comorbidities in adults that are associated with severe COVID-19, pre-existing immunity to other coronaviruses, and differences in melatonin levels (16–21). Nonetheless, mechanisms involved in milder COVID-19 disease in children are not fully understood.

The overall mortality rate in this study was 0.4%, which is similar to that reported in a meta-analysis by Badal et al. (0.3%, CI 95% 0.1–0.4) (20). However, the mortality rate of children who were hospitalized with COVID-19 in our cohort was 8.4%, which is significantly greater than that reported from the United States (1.8–5.2%) (22, 23) but lower than that seen in India (11.4%) (24) Indonesia (10.2%) (25) and Iran (13%) (19). These differences may be explained by variables such as predominant virus driving the outbreak at the time of reporting, social restriction measures enforced, prevalence of comorbidities, socioeconomic issues, health infrastructure, or even ethnicity as multisystem inflammatory syndrome has been more frequently reported in Hispanic children (26). We also hypothesize that the high prevalence of vitamin D deficiency in our country (27.3% in pre-school-age and 17.2% in school-age children) (27) could have contributed to COVID-19 mortality, as reported by Jayawardena R et al. (28).

Although Mexico City accounted for a majority of pediatric COVID-19 cases, CFR was lower compared to other States. This observation concurs with data from previous reports in the Mexican adult population (15) and with results from other studies that identified an inverse relation between death rate and population density (29–31). Additionally, this trend could be related to access to health care services, better infrastructure, availability of ICUs, and the impact of measures such as a social distancing, lockdowns, and immunizations.

Our analysis revealed differences in fatality rates among the health care services and also between the states. We surmise that socioeconomic inequalities could have contributed to higher mortality in our country, as reported by our colleagues in Mexico (26, 29) and Brazil (27, 32, 33). It is necessary to highlight the fact that these differences in case density did not coincide with higher frequency of hospitalization, admission to intensive care, or mortality. Therefore, such differences between states require a review of the social determinants of health, especially poverty index, transportation, and hospital infrastructure.

We show the perinatal period to be associated with a higher fatality rate than later childhood, indicating that the U-shaped COVID-19 mortality curve in the Mexican population is characterized by an initial decrease in mortality after 1 year of age, a nadir at 5–12 years, and a subsequent increase throughout life. This is similar to results reported by us previously in the same population (6, 15, 29), and the U-shaped mortality curve has been observed by others as well (5, 6, 34, 35). Worldwide, the highest mortality rate was seen in infants <1 year old, mainly in the lower and middle income countries (36), which lead to doubts about the usefulness of measures to control infection transmissibility in this age group as well as strategies to protect immunity during pregnancy and vaccinate pregnant women (37).

Risk factors associated with COVID-19 mortality identified here include a few that have been reported earlier, such as younger age (34, 37) and the presence of chronic conditions (19, 36–39). Further, our data show that a third of non-survivors had at least one comorbidity. Congruently, previous reports show that the presence of more than one pre-existing medical condition could increase the odds of death by up to 10-fold (16, 33, 40, 41). Some authors have proposed that the history of a comorbidity supersedes the effects of age, gender and race/ethnicity as risk factors for pediatric COVID-19 death (16), strongly advocating for vaccination of this vulnerable group (42).

Children with comorbidities had a higher mortality in our population, which concurs with other reports, including a meta-analysis that quantified mortality risk in children with underlying conditions (relative risk, 2.81, 95%CI 1.21–6.02). Further, obesity was associated with highest risk (RR 2.87, 95%CI 1.16–7.07) (41), and we report a HR of 1.92 (95%CI 1.5–2.4) for COVID-19 mortality due to obesity. Several studies have also found an association between obesity and general mortality, including in pediatric patients (29, 43–46). These observations are important given the high prevalence of overweight and obesity in the Mexican pediatric population (from 8.4% overweight children under 5 years of age to 43.8% joint prevalence of overweight and obesity in adolescents) (47).

Similar to observations in adults, diabetes was identified as another risk factor for mortality in this cohort (HR 2.0, 95%CI 1.4–2.8) (15). This finding is in agreement with reports from Iran (16) and with a previous report from Mexico (29). However, data from China, Italy, Spain, and United States suggest that children, adolescents, and youth with diabetes have similar COVID-19 outcomes compared to their non-diabetic-peers (47). These differences in trends could be explained by variation in metabolic control, access to health systems, and even differences in vaccination frequencies in this vulnerable population.

Among the comorbidities analyzed, immunosuppression had the highest CFR. This group includes oncology patients who suffer high mortality (48–50) or patients with primary immunodeficiency (51); however, other reports state that immunocompromised pediatric patients with cancer and hematopoietic cell transplant patients tended to experience clinical outcomes similar to that seen in the general population (52–54). Indirect effects of the pandemic, such as delayed treatment and the protection measures in this population, including the frequency and timing of immunization, may be contributing factors.

Other comorbidities associated with higher mortality in our study, such as chronic kidney disease (19, 29), cardiovascular disease (19) or hypertension (29), have also been identified in previous reports. Hence, children suffering from these diseases should be afforded vaccination priority, regardless of age. Although asthma was one of the most frequent comorbidities in this cohort (2.5%), it was not associated with greater mortality. This interesting finding provides a favorable outlook for asthmatic children infected by SARS-CoV-2 and is probably related to immune mechanisms (55).

The strengths of this study lie in the inclusion of all confirmed cases in Mexico using data from the national database. Further, stratification by variables that are directly related to death (hospitalization, pneumonia, and ICU) lowers the explanatory capacity of the models and demonstrates the need to include other undetermined variables such as the type of treatment. Notably, this strategy enables potential control of ecological fallacy bias; nevertheless, risks of bias inherent in retrospective studies are applicable here as well. As data was collected from reports that each hospital in the country provided daily, a memory bias cannot be excluded. Additionally, operational definitions used for exposure variables, such as comorbidities (mainly the variables of asthma, cardiovascular disease, and immunosuppression), were not standardized (risk of misclassification bias) and the follow-up strategy was different for each hospital (follow-up bias).

## Conclusion

Based on data available in the national database, we show that infant fatality rate due to SARS-CoV-2 in Mexico is similar to that reported in other countries. Vulnerable groups are patients younger than 12 months and those with comorbidities, and the distribution of mortality and access to health care showed heterogeneity across the country.

## Data availability statement

Publicly available datasets were analyzed in this study. This data can be found here: https://www.gob.mx/salud/documentos/datos-abiertos-152127.

## Ethics statement

Ethical review and approval were not required for the study on human participants in accordance with the local legislation and institutional requirements. Written informed consent was not provided because Research subjects were obtained from the public database of the Secretariat of Health of Mexico. Access to the identity of the research subjects is not possible.

## Author contributions

HM-G and MK-K organized the database and performed the statistical analysis. AM-L and FS-S wrote the first draft of the manuscript. AM-L, FS-S, HM-G, and MK-K wrote sections of the manuscript. All authors contributed to manuscript revision, read, and approved the submitted version.
