# Supplementary figures and images for: Survival analysis and mortality predictors of COVID-19 in a pediatric cohort in Mexico
Source: Front Public Health. 2022 Dec 16;10:969251. doi: 10.3389/fpubh.2022.969251 (PMC9801985; doi:10.3389/fpubh.2022.969251)

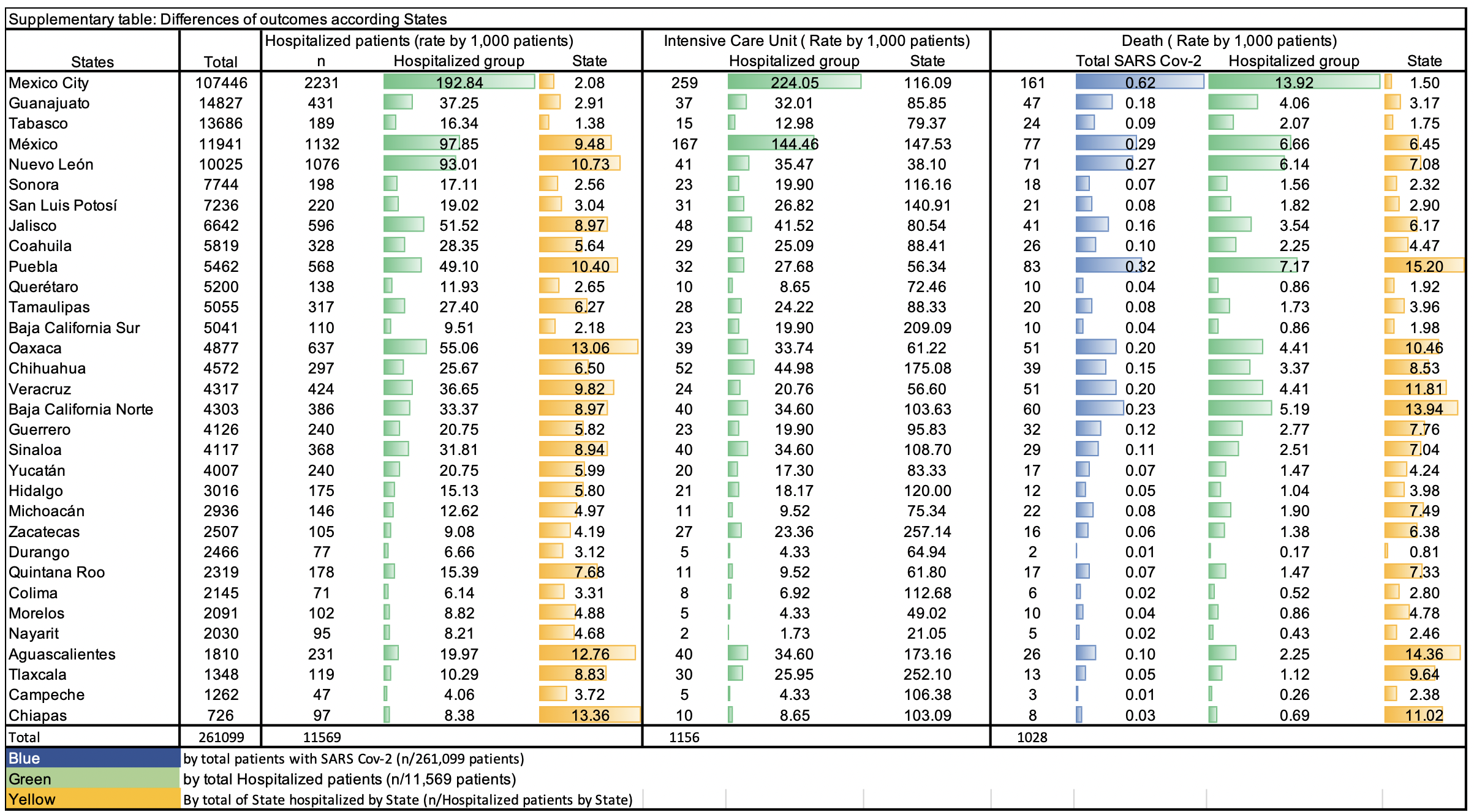

Supplement: Supplementary file 1 [file Table_1.DOCX]
